# Double-negative (CD27^−^IgD^−^) B cells are expanded in NSCLC and inversely correlate with affinity-matured B cell populations

**DOI:** 10.1186/s12967-018-1404-z

**Published:** 2018-02-15

**Authors:** Sara M. Centuori, Cecil J. Gomes, Samuel S. Kim, Charles W. Putnam, Brandon T. Larsen, Linda L. Garland, David W. Mount, Jesse D. Martinez

**Affiliations:** 1 University of Arizona Cancer Center, University of Arizona, 1515 N. Campbell Ave., Tucson, AZ 85724 USA; 2 Cancer Biology Graduate Interdisciplinary Program, University of Arizona, Tucson, AZ 85724 USA; 3 Department of Surgery, University of Arizona, Tucson, AZ 85724 USA; 4 Department of Laboratory Medicine and Pathology, Mayo Clinic, Scottsdale, AZ 85259 USA; 5 Department of Medicine, Division of Hematology/Oncology, University of Arizona, Tucson, AZ 85724 USA; 6 Department of Molecular and Cellular Biology, University of Arizona, Tucson, AZ 85724 USA; 7 Cell & Molecular Medicine, University of Arizona, Tucson, AZ 85724 USA

**Keywords:** TIL-Bs, B cells, CD27, NSCLC, Double-negative B cells, Lung cancer, Memory B cells

## Abstract

**Background:**

The presence of B cells in early stage non-small cell lung cancer (NSCLC) is associated with longer survival, however, the role these cells play in the generation and maintenance of anti-tumor immunity is unclear. B cells differentiate into a variety of subsets with differing characteristics and functions. To date, there is limited information on the specific B cell subsets found within NSCLC. To better understand the composition of the B cell populations found in NSCLC we have begun characterizing B cells in lung tumors and have detected a population of B cells that are CD79A^+^CD27^−^IgD^−^. These CD27^−^IgD^−^ (double-negative) B cells have previously been characterized as unconventional memory B cells and have been detected in some autoimmune diseases and in the elderly population but have not been detected previously in tumor tissue.

**Methods:**

A total of 15 fresh untreated NSCLC tumors and 15 matched adjacent lung control tissues were dissociated and analyzed by intracellular flow cytometry to detect the B cell-related markers CD79A, CD27 and IgD. All CD79A^+^ B cells subsets were classified as either naïve (CD27^−^IgD^+^), affinity-matured (CD27^+^IgD^−^), early memory/germinal center cells (CD27^+^IgD^+^) or double-negative B cells (CD27^−^IgD^−^). Association of double-negative B cells with clinical data including gender, age, smoking status, tumor diagnosis and pathologic differentiation status were also examined using the logistic regression analysis for age and student’s t-test for all other variables. Associations with other B cell subpopulations were examined using Spearman’s rank correlation.

**Results:**

We observed that double-negative B cells were frequently abundant in lung tumors compared to normal adjacent controls (13 out of 15 cases), and in some cases made up a substantial proportion of the total B cell compartment. The presence of double-negative cells was also found to be inversely related to the presence of affinity-matured B cells within the tumor, Spearman’s coefficient of − 0.76.

**Conclusions:**

This study is the first to observe the presence of CD27^−^IgD^−^ double-negative B cells in human NSCLC and that this population is inversely correlated with traditional affinity-matured B cell populations.

## Background

The tumor microenvironment is a complex dynamic network of stromal components, fibroblasts, endothelial cells, stem cells, and infiltrating immune cells, all of which play roles in tumor development and progression, hence patient prognosis. Consistent with this paradigm, various sub-classes of tumor-infiltrating T cells exhibit strong associations with patient survival in a variety of human malignancies [1]. Tumors may also be densely infiltrated with B cells, yet their role in anti-tumor immunity is far less understood [2]. Several reports investigating the role of B cells in the context of non-small cell lung cancer (NSCLC) have shown an upregulation of B cell related genes [3], CD20^+^ B cell infiltration [4], and the presence of B cell-related tertiary lymphoid structures [5] to correlate with patient survival, suggesting that B cells may have influential roles in lung tumor immunity. This supposition is however challenged by data suggesting that humoral immune responses may instead attenuate T cell mediated tumor immunity [6]. These seemingly contradictory observations reinforce the importance of dissecting the role of B cells in anti-tumor immunity. To that end, we sought to examine the functional states of B cells within NSCLC tumors, based upon their phenotypic characteristics, i.e. their expression of cell surface markers.

In the course of our efforts to characterize the intra-tumoral B cell subsets in NSCLC, we detected a previously poorly-characterized population of CD79A^+^CD27^−^IgD^−^ B cells (CD79A is a pan-B cell marker) [7, 8]. B cells lacking CD27 and IgD have recently been termed double-negative memory B cells (DN B cells). It has been demonstrated that these cells can be detected in heathy individuals at low levels within peripheral blood and tonsils [7, 9], but are expanded in peripheral blood of elderly patients [10], patients with Alzheimer’s disease [11], rheumatoid arthritis [12], systemic lupus erythematosus [7], HIV [13], and rotavirus [8].

It has been suggested that DN B cells, unlike memory B cells which are CD27^+^, are incapable of acting as antigen presenting cells (APCs) due to their low levels of CD80 and CD40, as well as lacking the stimulatory molecule IL-6R [10] and display more inhibitory molecules (CD330a and FcRH4) [8, 10]. In addition, levels of hypermutation have also been evaluated and compared against traditional memory B cell phenotypes. Reports demonstrate that DN B cells display a degree of hypermutation that is significantly lower than that of their conventional CD27^+^ class-switched counterparts and similar to levels observed on CD27^+^IgD^+^ non-switched memory cells [7, 8, 14].

B cell maturation typically occurs in germinal centers within lymph nodes and other secondary lymphoid tissues where they undergo antibody affinity maturation and differentiation. Germinal center reactions produce highly specific plasma or memory B cells that have acquired affinity to a specific antigen [15]. IgD and IgM are the earliest immunoglobulins expressed by B cells and form the B cell receptor (BCR), which binds antigen [16]. Current models hold that the absence of IgD on B cells indicates that the cell has been class-switched, that is, activation and proliferation had been triggered by the antigen-BCR interaction [17]. This is true in most cases except for IgM only cells, a rare B cell subset that expresses high levels of IgM but low or absent levels of IgD and are not class-switched. IgM only cells are also CD27 positive and therefore are considered memory B cells, typically representing less than 5% of peripheral blood B cells [14]. The process of IgD downregulation is followed by an almost immediate upregulation of the CD27 molecule. CD27, a member of the TNF-R superfamily, forms part of the BCR complex that assists in the propagation of activation signals [18]. CD27 is expressed globally on germinal center B cells and early memory B cells (CD27^+^IgD^+/−^) found in secondary lymphoid organs, 15–20% of peripheral B cells in the blood (CD27^+^IgD^+^), as well as B cells that have undergone class switching (CD27^+^IgD^−^) [19–21]. CD27 negative cells are considered naïve and therefore are unswitched, maintaining their expression of IgD (CD27^−^IgD^+^). Loss of IgD expression on double-negative (CD27^−^IgD^−^) B cells indicates that they have undergone class switching, though they do not gain the expression of CD27. Little is known about what consequences the lack of CD27 has on the DN B cell population. Furthermore, even less is known about how this DN B cell population effects tumor immunity.

In this study we characterized the various B cell populations in untreated NSCLC tumors and matched normal controls. During our investigation we observed that these samples harbor, to varying degrees, DN B cells and in many cases these cells comprised a significant proportion of the total B cell population. Furthermore, we documented an inverse correlation of DN B cells with the affinity-matured B cell population.

## Methods

### Collection of human tissues

Fresh surgically resected human NSCLC tumor samples and normal adjacent lung tissues (matched normal controls) were obtained from the UACC TACMASR collection service and biorepository. All samples were obtained from untreated patients in accordance with TACMASR IRB approval and patient consent. Tissue adjacent lung samples were collected and served as matched normal controls. Samples were de-identified in accordance with our IRB protocol.

### Dissociation of human lung tissues

Tissues were first mechanically then enzymatically dissociated. Using a p10 scalpel, tissues were cut into 0.1 mm^3^ sections and transferred into a clean 50 ml tube. Freshly prepared cell dissociation solution (10 mls) containing 1 mg/ml Collagenase A (Sigma), 0.5% Trypsin (Corning), 100 U/ml DNase 1 (Sigma) and 5 mM magnesium chloride (Sigma), in FBS-free RPMI was used to enzymatically dissociate samples. Tissue samples were incubated at 37 °C on an orbital shaker at 100 RPM for 45 min. Then the reaction was neutralized using 5 ml/tube of FBS. The cell suspension was then passed through a 100 μM cell strainer to obtain single cell suspensions.

### Intracellular flow cytometry

Single cell suspensions were washed with PBS and 1.0 × 10^6^ cells/tube were FC blocked (eBioscience) and stained extracellularly for 30 min at 4 °C. Cells were then washed and prepared for intracellular staining using the FoxP3 Intracellular Staining Kit (R and D systems). Intracellular staining was performed at 4 °C for 60 min. Antibodies CD79A-APC, CD27-FITC, IgD-PE, CD4-APC, CD45RO-PE, CD8-FITC, and CD3-APC were purchased from eBioscience.

### Statistical analysis

Logarithm transformation was applied to normalize the percentage of DN B cells. Comparisons of the percentage of DN B cells between tumor and matched normal lung samples were made with the paired student’s t-test. To evaluate all other categorical variables unpaired student’s t-tests were performed. Logistic regression analysis was used to analyze the continuous variable age. Spearman’s rank correlation was performed to evaluate associations between various B cell populations.

## Results

### The double-negative (CD79A^+^CD27^−^IgD^−^) B cell population is expanded in NSCLC tumors

Previously it has been reported that the expression of B cell-related genes (2) and the presence of tumor infiltrating B cells (TIL-Bs) (4) correlate with early stage lung cancer survival. Accordingly, we characterized TIL-Bs in untreated NSCLC tumors and matched normal lung tissue to identify B cell subsets which might participate in anti-tumor immunity. To do so, tumors and adjacent normal lung tissue samples were dissociated into single cells, stained with a panel of B cell-related antibodies and analyzed by flow cytometry. We consistently identified a population of CD79A^+^CD27^−^IgD^−^ B cells, also known as double-negative B cells, a rare subset of B cells not previously characterized in malignant tumors [22].

**Fig. 1:**
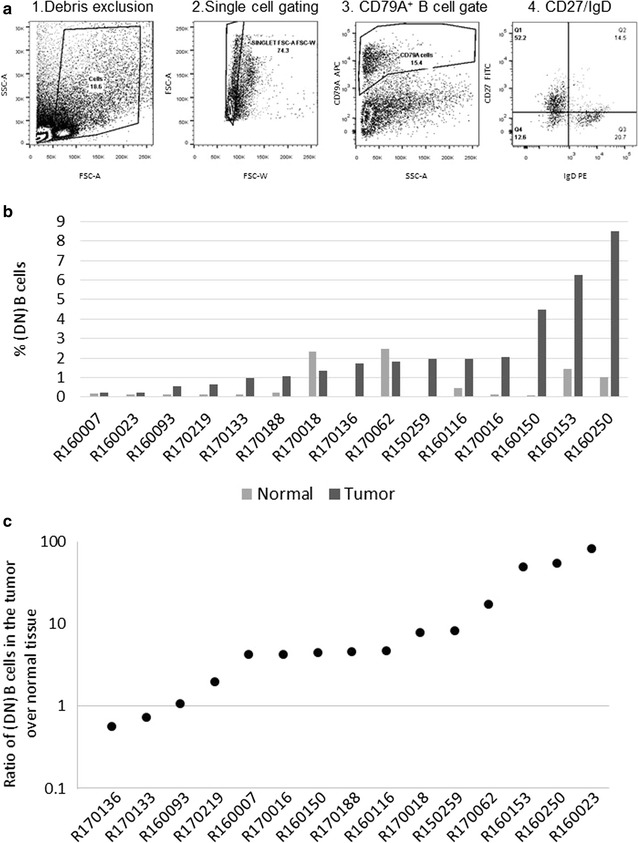
DN B cells are expanded in NSCLC tumors. Single cell suspensions of NSCLC tumors and matched normal lung tissue samples were stained with APC-CD79A, PE-IgD and FITC-CD27. **a** Schema for the enumeration of CD27^−^IgD^−^ DN B cells by flow cytometry (1) debris is excluded based on FCS-A, SSC-A, (2) single cells are selected based on FSC-W and FCS-A (3) then the total B cell population is gated based on APC-CD79A expression (4). Finally, the percentage of DN B cells were defined by the absence of PE-IgD and FITC-CD27 expression. **b** Shown is the percentage of CD79A^+^CD27^−^IgD^−^ DN B cells present out of total live cell singlet population analyzed by flow cytometry of tumor versus normal lung for each patient studied. The cases are arranged in ascending order according to the percentage of DN B cells within the tumor. **c** Ratio of DN B cells was calculated by dividing the percentage of DN B cells found within the tumor by that found within the matched normal tissue for each patient. Values are arranged in ascending order. Paired students t-test was used to evaluate the statistical differences between tumor and matched normal control tissues. *p < 0.001

To quantify the DN B cell population, single cells were gated by the schema illustrated in Fig. 1a; additionally, accurate quadrant placement for each sample was verified by back gating strategies allowing for the identification of true CD27 negative and IgD negative population locations. The DN B cell population was detectable in all 30 patient tissues examined (15 NSCLC tumors and 15 adjacent normal lung tissues). The proportion of DN B cells (of total live singlet cells analyzed) for each sample is shown in Fig. 1b; the values ranged between 0.2–8.5% in tumors and 0.02–2.5% in the normal lung tissues. Moreover, in 13 of the 15 patients, the ratio of DN B cells in the carcinoma compared to the adjacent normal lung sample was greater than one (Fig. 1c), suggesting the frequent expansion of the DN B subset in NSCLC tumors. This expansion was tested using the paired students t-test and was found to be significant (p value < 0.001).

### Clinical parameters associated with the presence of DN B cells

We sought to correlate the extent of the DN B cell subset in tumors with the available clinical data, including gender, age, smoking status, tumor diagnosis and pathologic differentiation status, as summarized in Table 1. The levels of DN B cells were significantly (p = 0.04) higher in patients with moderately differentiated tumors versus those with poorly differentiated tumors; otherwise, no significant correlations with clinical attributes could be identified (Table 2). We also analyzed the relationship of DN B cells in the normal tissue samples in relation to smoking status (p = 0.923) and gender (p = 0.698), but again no significant trends were observed (data not shown).

**Table 1:** Clinical information associated with NSCLC patient cohort

Patient ID	Diagnosis	Age	Smoking status	Gender	Stage	Differentiation	% DN cells out of total live singlets
R160007	SqCC	70	Smoker	Female	Stage IB	Poorly differentiated	0.204
R160023	Adeno	86	Non-Smoker	Female	Stage IIB	Poorly differentiated	0.222
R160093	Sqcc	69	Smoker	Male	StageIB	Moderately differentiated	0.536
R170219	Sqcc	69	Smoker	Male	Stage IIIA	Poorly differentiated	0.643
R170133	Sqcc	62	Non-Smoker	Female	Stage IB	Poorly differentiated	0.985
R170188	Adeno	72	Smoker	Female	Stage IA	Poorly differentiated	1.088
R170018	Sqcc	74	Smoker	Female	Stage IIIA	Poorly differentiated	1.341
R170136	Sqcc	60	Smoker	Female	Stage IIIA	Moderately differentiated	1.721
R170062	Sqcc	83	Smoker	Male	Stage IB	Moderately differentiated	1.830
R150259	Sqcc	75	Smoker	Male	Stage IB	Poorly differentiated	1.943
R160116	Sqcc	73	Smoker	Male	Stage IIB	Poorly differentiated	1.969
R170016	Adeno	71	Non-Smoker	Female	Stage IB	Poorly differentiated	2.059
R160150	Adeno	71	Smoker	Female	Stage IB	Moderately differentiated	4.471
R160153	Adeno	84	Non-Smoker	Female	Stage IB	Moderately differentiated	6.269
R160250	Adeno	84	Non-Smoker	Male	Stage IIA	Moderately differentiated	8.492

Smoking status was defined as smoker including the groups current and former smoker and non-smoker including the groups non-smoker and never smokers

*SqCC* squamous cell carcinoma, *Adeno* adenocarcinoma

**Table 2:** Association between % (DN) B cells in NSCLC tumors and clinical parameters

	n	Mean (SE)	p value
Diagnosis			
SqCC	9	1.24 (0.68)	0.17
Adeno	6	3.76 (0.84)	
Smoking status			
Smoker	10	1.63 (0.77)	0.48
Non-smoker	5	3.60 (1.03)	
Gender			
Male	6	2.57 (1.02)	0.65
Female	9	2.16 (0.89)	
Stage			
I	9	2.28 (0.88)	0.94
II	3	3.56 (1.44)	
IIIA	3	1.23 (1.43)	
Differentiation			
Poorly differentiated	9	1.16 (0.66)	0.04*
Moderately differentiated	6	3.88 (0.81)	

* p < 0.05

Because previous studies have shown that the double-negative subset is expanded in the peripheral blood of the elderly [8–10, 23, 24], a possible influence of age on the presence of DN B cells was explored using linear regression analysis of data collected from either the NSCLC tumors or normal lung tissues. Consistent with published data from peripheral blood samples, there was a statistically significant correlation (p = 0.002) between increasing age and the proportion of DN B cells in normal lung tissue presenting with an estimate coefficient of 0.17 and a standard error of 0.04. Although elevated levels of DN B cells in older patients persisted in the NSCLC samples, this trend did not reach statistical significance (p = 0.06), with an estimate coefficient of 0.08 and a standard error of 0.03.

### The size of the DN B cell subset is inversely correlated with the affinity-matured B cell population

**Fig. 2:**
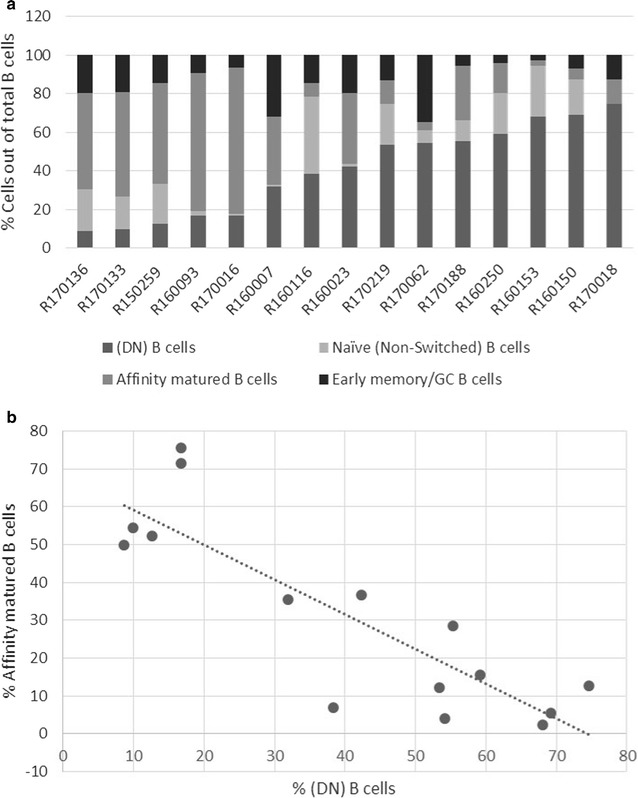
DN B cells are inversely correlated with the presence of affinity-matured B cells. **a** Tumor samples were first gated for single cells and then for APC-CD79A expression. The percentage of each subset within this population is defined by PE-IgD and FITC-CD27 expression as follows: CD27^−^IgD^−^ (DN) B cells, CD27^+^IgD^−^ Affinity-matured B cells, CD27^−^IgD^+^ Naïve (non-switched) B cells, and CD27^+^IgD^+^ Early memory/GC (germinal center) B cells. The data are presented according to ascending amounts of DN B cells. **b** The percent of CD27^−^IgD^−^ DN B cells (x-axis) and percent of CD27^+^IgD^−^ Affinity-matured B cells (y-axis) were defined as described above and plotted for each patient in ranked order. Spearman’s rank correlation coefficient was determined to be − 0.76 with a p-value of 0.001

Next, we sought to identify possible relationships between the double-negative population and the three other B cell subsets present within the tumor microenvironment. To that end, we gated on CD79A^+^ B cells and compiled the percentages of DN B cells (CD27^−^IgD^−^), naïve B cells (CD27^−^IgD^+^), affinity-matured B cells (CD27^+^IgD^−^), and early memory/germinal center (GC) B cells (CD27^+^IgD^+^) (Fig. 2a) [25]. Tumors harboring larger DN B cell populations had fewer affinity-matured B cells; a comparison between the two populations confirmed an inverse relationship (Spearman’s rank correlation coefficient, − 0.76, p = 0.001) (Fig. 2b). Additionally, we examined associations between the DN B cell population and all other B cell subsets and found no significant relationships (data not shown). These data suggest a possible etiologic relationship between the relative numbers of DN and affinity-matured B cells within the tumor microenvironment.

## Discussion

Here we provide data indicating the presence, in both NSCLC tumors and normal lung tissue, of CD27^−^IgD^−^ (double-negative) B cells, a subset of B cells not previously described in malignant tumors. We analyzed by flow cytometry 15 NSCLC tumors together with 15 adjacent normal lung samples for the presence or absence of CD27 and IgD. In the normal lung samples, DN B cells ranged from 0.02 to 2.5% and were positively correlated with age, mirroring previous reports documenting similar findings in the peripheral blood. Although the DN B cell population in tumor samples also appeared to correlate with age, this trend failed to reach statistical significance. More importantly, in all but two patients, the proportion of DN B cells in the tumor samples were significantly greater than in the corresponding normal lung tissue, indicating expansion of this B cell subset in NSCLC tumors. This report is the first to reveal a tumor-specific expansion of this otherwise rare B cell population.

The percentages of DN B cells in the NSCLC tumors varied widely, between 0.2 and 8.5%. Other than the differentiation status of the tumor—moderately differentiated tumors exhibited higher concentrations of DN B cells than poorly differentiated ones—we could not identify associations with the other clinical parameters examined. An analysis of survivorship in relationship to DN B cell status must of course await further long-term follow-up studies.

Perhaps our most striking finding is that of an inverse correlation between DN B cells and affinity-matured B cells. In affinity-matured B cells, CD27 is upregulated while IgD is downregulated; whereas in DN B cells the downregulation of IgD is accompanied by the absence of CD27. Lack of CD27 on a specific subset of exhausted B cells has recently been described in NSCLC [26]. Their presence has been associated with a reduction in the ability of TIL-B cells to present tumor antigen and positively correlated with the presence of regulatory T cells (Tregs); suggesting the lack of CD27 on TIL-B cells may hinder effective anti-tumor immunity. Although these studies on exhausted B cells did not examine the presence of IgD for class switching, it is possible that the CD27^−^IgD^−^ double-negative cells in this study may have similar functional characteristics.

Unlike exhausted B cells, it remains unclear how DN B cells arise; at least four mechanisms have been suggested: (1) DN B cells are exhausted memory B cells that consequently exhibit downregulated CD27; (2) they are the product of a defective germinal center reaction in which CD27 fails to upregulate appropriately; (3) they represent precursor memory B cells that have yet to upregulate CD27; (4) they represent a unique B cell subset, possibly formed by a germinal center-independent process. Traditional CD27^+^ memory B cells initially express IgM and IgD and are then class-switched to express immunoglobulin isotypes IgG, IgA, or IgE. Previously, CD27 was considered to be an all-encompassing memory B cell marker which accounted for all class-switched subsets [19, 27]. However, further research elucidated that between 10 and 20% of IgG^+^ class-switched memory B cells were CD27^−^ [28]; leading to postulation of the existence of CD27^−^ memory cells, i.e., DN B cells. In the same study, a large proportion of IgG^+^CD27^−^ cells harbored BCL6 mutations indicative of germinal center derivation. While origins of the collective DN B cell population remain uncertain, it is plausible that a fraction of this population is derived from IgG^+^CD27^+^ germinal center memory B cells. In further support of this, some DN B cells from peripheral blood have been shown to share a high frequency of clonally related sequences with both traditional memory B cell subsets and marginal zone B cells, leading to the supposition of a common lineage [25]. Although our data demonstrating an inverse relationship between DN and affinity-matured B cells does not favor one hypothesis over another, it does suggest the possibility that the generation of DN B cells is at the expense of the affinity-matured B cell population, which are critical to the generation of an immune response.

## Conclusions

This study revealed the presence of a CD27^−^IgD^−^ B cell subset in untreated NSCLC and demonstrated that their presence inversely correlates with the presence of affinity-matured B cell populations from the same patient.

These findings are impactful and significantly contribute to the field, since to date, double-negative B cells have not previously been described in the context of cancer. B cells are a positive predictive indicator in NSCLC as well as in other solid tumor, however, there is very little known about what specific subsets contribute to this effect. The observation that double-negative B cells exist in NSCLC tumors and make up a large proportion of the total B cell compartment provides preliminary evidence that their presence may influence tumor immunity. Furthermore, the fact that expansion of this population negatively correlated with the presence of other affinity-matured B cell populations provides further evidence that these cells should be taken into consideration when evaluating the immune landscape in cancer.

## Abbreviations

APCs: antigen presenting cells
DN: double-negative
GC: germinal center
NSCLC: non-small cell lung cancer
TIL-Bs: tumor infiltrating B cells
Tregs: T regulatory cells
